# Recovery Capital and Family Support Among Incarcerated Men with a History of Psychoactive Substance Use in a Single Romanian Penitentiary: A Qualitative Study

**DOI:** 10.3390/healthcare14172873

**Published:** 2026-09-07

**Authors:** Cornelia Rada, Maria-Miana Dina, Cristina-Roxana Iureși

**Affiliations:** 1 Biomedical Department, Francisc I. Rainer Institute of Anthropology, Romanian Academy, Academy House, 13 September Avenue, No. 13, 5th District, 050711 Bucharest, Romania; 2 Directorate of Social Reintegration, National Administration of Penitentiaries, 39 Calea Floreasca Street, 023761 Bucharest, Romania; grebenar.miana@yahoo.com; 3 School of Advanced Studies of the Romanian Academy, “Constantin Rădulescu-Motru” Institute of Philosophy and Psychology, Department of Psychology, Romanian Academy, Academy House, 13 September Avenue, No. 13, 5th District, 050711 Bucharest, Romania; asociatia.fluens@yahoo.com

**Keywords:** substance use disorders, prison health, recovery capital, family support, qualitative research, social reintegration, incarceration, public health, addiction recovery, recidivism

## Abstract

**Highlights:**

- Illicit drug use was predominantly initiated under the influence of peer groups, criminogenic environments, and socioeconomic vulnerabilities.
- Abstinence achieved during incarceration was perceived by part of this sample as maintained under institutional constraint rather than as internally motivated recovery.
- The family functioned as both a risk and a protective factor; the quality, not merely the presence, of support distinguished protective from inadvertently facilitating family environments.
- Family support, occupational integration, and prosocial relationships were resources participants associated with periods of abstinence, alongside a recurring cognitive pattern of externalizing responsibility for use onto peers or circumstance.

**Abstract:**

**Background/Objectives:** Substance use disorders are common in the prison population and are associated with relapse and criminal recidivism, yet qualitative evidence on recovery capital and family support within Eastern European penitentiary systems remains limited. This study explored how incarcerated men with a history of substance use perceive initiation, withdrawal, recovery, and the role of family support as a recovery capital resource. **Methods:** A qualitative descriptive study was conducted in a Romanian penitentiary with 50 definitively sentenced men with self-reported substance use history, selected through purposive sampling. Data were collected through semi-structured interviews, without audio recording due to institutional security regulations, and reconstructed in detailed written notes validated through member checking, then analyzed using inductive thematic analysis via consensus-based double coding (initially coded by the first researcher and reviewed by a second researcher, with discrepancies resolved through discussion), until thematic saturation was reached. **Results:** Six themes were identified: initiation of use influenced by peer groups, socioeconomic vulnerability, and criminogenic environments; effects initially perceived as beneficial, followed by progressive deterioration; severe withdrawal and denial of dependence; cognitive mechanisms sustaining use, including externalization of responsibility; the dual role of the family as both a risk and a protective factor; and a recovery process marked by ambivalence toward abstinence, in which family support, employment, and prosocial relationships were central. Abstinence during incarceration was frequently perceived as maintained under institutional constraint rather than as internally motivated recovery. **Conclusions:** In this single-prison sample, recovery extended beyond abstinence, suggesting the need to strengthen personal, social, and family recovery capital. The findings, which require confirmation in larger samples, support exploring integrated prison–community continuum-of-care interventions centered on addiction treatment, family involvement, and post-release support.

## 1. Introduction

According to the World Health Organization and the World Drug Report of the United Nations Office on Drugs and Crime (UNODC), psychoactive substance use represents a major global public health problem, with a consistently increasing trend over the past decades [1,2]. In 2024, an estimated 331 million people used drugs, a 34% increase compared to a decade earlier. Cannabis remains the most commonly used substance (256 million users), followed by opioids, amphetamines, cocaine, and 3,4-methylenedioxymethamphetamine (MDMA) [2].

Terminological note: this manuscript uses “psychoactive substance use” and “illicit drug use” as largely overlapping but not fully synonymous terms—the former is the broader public-health category used in international reporting (e.g., WHO, UNODC), while the latter, used mainly in Section 2, Section 3 and Section 4, reflects that all substances reported by this specific sample were used illicitly, consistent with the legal status of these substances in Romania. When referring to individuals, the manuscript consistently uses “individuals/persons with a history of substance use” rather than “drug users” or “addicts,” except within direct quotations or when citing terminology used by a specific external source.

At the European level, data indicate a high prevalence of psychoactive substance use, particularly cannabis and stimulants, with a concerning increase in cocaine use in recent years [3]. At the same time, the global burden of disease associated with alcohol and drug use remains high, correlated with premature mortality, disability, and significant socioeconomic costs [4,5,6].

From a public health perspective, addiction is conceptualized as a chronic, relapsing disorder with biological, psychological, and social determinants, characterized by alterations in the neurobiological circuits involved in impulse control and decision-making [7]. This approach is supported by contemporary literature in neuroscience and addiction psychiatry, which treats addiction as a complex condition with a long-term course and an elevated risk of relapse [6,8].

In this context, substance use disorders are associated with a major impact on public health, including overdose, psychiatric comorbidities, transmission of infectious diseases, and increased mortality [1]. At the same time, access to treatment remains limited, with a substantial “treatment gap” reported globally, in which only a small proportion of affected individuals receive adequate interventions [2].

Beyond its medical dimension, psychoactive substance use is closely associated with antisocial and criminal behavior. Meta-analyses indicate a significant association between substance abuse and criminal behavior and recidivism [9,10,11].

A history of violence is frequently observed among individuals in addiction treatment [12]. Under the influence of psychoactive substances, alterations in inhibitory control, increased impulsivity, diminished capacity to evaluate consequences, and impaired social judgment may occur—factors that can facilitate involvement in aggressive or unlawful behavior [13].

In particular, the use of certain substances has been associated with an increased risk of violent behavior, interpersonal conflict, and decisions with criminal potential, especially in contexts marked by social vulnerability, trauma exposure, and insufficient family support [12,14].

Recent research on young people involved in the criminal justice system indicates that substance use is one of the main risk factors for antisocial behavior and recidivism [15].

Furthermore, substance use is also correlated with non-violent offenses, often driven by the need to finance addiction, thereby reinforcing the use–crime cycle [16]. From the perspective of the risk–needs–responsivity model, substance use represents a central criminogenic factor associated with recidivism and difficulties in social reintegration [10,17]. Accordingly, incarcerated individuals constitute a population with heightened vulnerability, as the prevalence of substance use disorders is significantly higher compared to the general population [10,18].

The specialized literature emphasizes the need for integrated interventions combining medical, psychosocial, and criminal justice perspectives to reduce the impact of substance use on public health and social safety [1,6]. In recent years, researchers’ attention has increasingly turned to the concept of recovery capital, defined as the totality of personal, family, social, and community resources that facilitate the initiation and maintenance of recovery from addiction [19,20]. Studies have shown that functional family relationships, social support, and integration into prosocial networks are associated with a higher likelihood of maintaining abstinence, better quality of life, and reduced risk of recidivism [21,22].

In the specific context of Romania, national data indicate a concerning situation regarding psychoactive substance use. According to the National Report on the Drug Situation, prepared by the National Anti-Drug Agency [23], 10.7% of individuals aged 15 to 64 reported lifetime experimental use of illicit drugs, and 6% reported recent use. The highest prevalence is recorded among the young population (15–34 years), with adolescence representing the period of greatest vulnerability for initiation of use, and a minimum reported age of onset of 13 years for new psychoactive substances. Romania also stands out for a high prevalence of new psychoactive substance use compared to the European average, with cannabis and legal-high substances (“ethnobotanicals”) ranking among the most frequently used substances [23,24,25]. From a public health perspective, problematic substance use in Romania is associated with a significant rate of HIV and hepatitis C infections among people who inject drugs, further underscoring its status as a complex public health problem [23].

Despite this epidemiological reality, qualitative research on psychoactive substance use among the incarcerated population in Romania remains limited. Available national studies focus predominantly on quantitative epidemiological data or on outpatient clinical populations, without capturing the subjective perspectives of incarcerated individuals regarding trajectories of use, recovery mechanisms, and the role of family support as a recovery capital resource.

Regarding the services available in Romanian penitentiaries, official EUDA data indicate the presence of relevant continuum-of-care components—such as continuity of opioid agonist treatment (OAT) between the community and the penitentiary, therapeutic communities, and linkage to treatment and social assistance services—yet significant gaps persist in harm reduction interventions (e.g., needle and syringe programs, naloxone distribution) and in linkage to care for use-associated infectious diseases (HIV, HCV) [25]. At the same time, the post-release transition remains a critical stage that is institutionally under-addressed, with limited resources for continuity of care in the period immediately following release. It is important to note that EUDA data reflect the formal, administrative availability of these services, without providing information on how incarcerated individuals perceive, access, or effectively benefit from these interventions. The present study seeks to address this gap by offering a qualitative, insider perspective on the subjective experience of incarcerated individuals regarding the support resources they have benefited from or considered available, both prior to and during incarceration. A distinctive element of this research is its emphasis on family support as a complementary recovery capital resource—a dimension insufficiently documented in the Romanian specialized literature, yet directly relevant to understanding the recovery and social reintegration processes of this vulnerable population.

Qualitative and mixed-methods evidence on substance use among incarcerated populations exists internationally, including multi-country European work on prevalence and correlates of use during imprisonment [18] and systematic reviews of factors associated with in-prison drug use [26]; however, this literature rarely combines initiation, withdrawal, cognitive attribution of responsibility, and family support as a recovery-capital resource within a single qualitative account, and, to our knowledge, no comparable qualitative study has been conducted within a Romanian or, more broadly, Eastern European penitentiary system—even recent parallel qualitative work identifying family support as a key protective factor among incarcerated men, such as a 2025 study from Indonesia [27], comes from other, non-European collectivist contexts. The present study’s novelty therefore lies in this combined focus and in the national context, rather than in the individual thematic components taken separately.

Grounded in this theoretical framework, the study seeks to identify how individuals with a history of psychoactive substance use perceive and mobilize different forms of recovery capital along their trajectory—from the initiation of use, through the experience of incarceration, to their perspectives on post-release life. Particular attention is given to the dual function of the family as a potential source of both protection and vulnerability, as well as to its implications for the risk of criminal recidivism.

The general objective of the study was to explore the experiences of incarcerated individuals with a history of psychoactive substance use, with an emphasis on the factors associated with the initiation and maintenance of use, the recovery process, and the role of family support as a recovery capital resource in the context of post-release social reintegration.

### Specific Objectives

- To explore the social, contextual, and individual factors associated with the initiation of psychoactive substance use, with an emphasis on the specific vulnerabilities of early onset and exposure to criminogenic environments.
- To examine participants’ subjective experiences of the effects of substance use, dependence, and withdrawal, including the impact on psychological and behavioral functioning.
- To analyze the cognitive and social mechanisms sustaining addictive behavior, including patterns of responsibility attribution and the relationship between substance use and criminal behavior.
- To investigate the role of the family as a protective factor and/or risk factor along the trajectory of substance use, within the theoretical framework of the recovery capital concept.
- To identify factors perceived as facilitators of or barriers to abstinence and recovery in the prison environment, including relevant institutional and relational resources.
- To examine participants’ perspectives on post-release social reintegration, including factors associated with the risk of recidivism and community reinsertion.

## 2. Materials and Methods

### 2.1. Study Design

The study employed a qualitative descriptive design (qualitative description [28,29]), aimed at investigating the experiences of incarcerated individuals with a history of psychoactive substance use prior to incarceration.

A qualitative descriptive design is recommended when the research aim is to obtain a rich, comprehensive description of a lived phenomenon that remains as close as possible to the raw data, without adopting the epistemological commitments specific to a particular qualitative tradition (e.g., phenomenological bracketing or individual-case narrative reconstruction), and is suitable for samples larger than those typical of other qualitative designs [28,29,30]. This methodological choice is appropriate for the objectives of the present study, which aims at a comprehensive description of the subjective experience of substance use, incarceration, and the recovery process across the entire sample, including the reporting of summary quantitative sample characteristics (quasi-statistics) alongside the qualitative content itself [29].

This research is situated within the public health paradigm applied to vulnerable populations, in which substance use disorders are understood as chronic conditions with a multidimensional impact on individual and social functioning, including criminal behavior [1].

The reporting of this study followed the Consolidated Criteria for Reporting Qualitative Research (COREQ [31]), a reference guideline for qualitative studies in the health field.

Throughout this manuscript, “recovery” is used, consistent with the recovery capital framework [20], to denote a multidimensional process encompassing abstinence, engagement with therapeutic or support resources, reconstruction of personal identity, and progress toward social reintegration, rather than any single one of these elements in isolation; where a specific dimension (e.g., abstinence, identity reconstruction) is intended, it is named explicitly.

### 2.2. Setting and Participants

The study included 50 incarcerated men, definitively sentenced and held in the custody of Bucharest–Rahova Penitentiary, Romania. All participants had a history of psychoactive substance use prior to incarceration, according to information recorded in the penitentiary’s information system, based on inmates’ self-reports; this system records a self-reported history of use rather than a clinical diagnosis or standardized screening result. Accordingly, throughout this manuscript, terms such as “substance use disorder,” “dependence,” and “addiction” are used descriptively, to characterize participants’ reported experiences and the constructs discussed in the cited literature, and do not imply that a formal diagnosis was established for any participant. Sampling was purposive, aiming to maximize the diversity of experiences related to participants’ use trajectories and life courses. Recruitment followed an open-invitation procedure: an invitation to participate was extended to eligible incarcerated men, and enrollment continued until the target of 50 participants was reached, at which point recruitment was closed. Consequently, a conventional recruitment flow (total number invited versus those who declined) was not applicable to this quota-based procedure and was not recorded. Participants came predominantly from urban areas and showed variability in age and socioeconomic status, allowing for a comprehensive exploration of the phenomenon under investigation.

Inclusion criteria were as follows: self-reported substance use history, recorded in the penitentiary’s information system at the time of incarceration; a definitive sentence and detention within the Romanian penitentiary system; self-reported abstinence of at least 30 days at the time of the interview. Exclusion criteria were as follows: presence of acute psychotic symptoms, inability to provide informed consent, and recent violent behavior documented in the prison environment. A minimum self-reported abstinence period of 30 days was required to ensure that participants could provide reflective accounts of their experiences while minimizing the potential influence of acute intoxication or withdrawal symptoms, which may affect cognitive functioning and recall [7].

This methodological choice, however, also has a structural consequence for the sample: the minimum abstinence criterion excluded from the outset individuals with active use or recent relapse during incarceration, thereby selecting a subgroup characterized by the capacity or motivation to maintain short-term abstinence. The implications of this selection for the interpretation of the findings on abstinence are discussed in Section Limitations.

### 2.3. Data Collection

Data were collected during the fourth quarter of 2022 through individual semi-structured interviews. Interviews were conducted in designated spaces within the penitentiary, in compliance with institutional rules on safety and research ethics. Only the participant and the interviewer were present during each interview. Interview duration ranged from 45 to 60 min, depending on the depth of participants’ accounts and their availability.

Interviews followed a semi-structured guide comprising open-ended questions organized around five thematic dimensions: sociodemographic data and general context; the experience of substance use; attribution of responsibility for the onset of use; periods of abstinence and associated factors; and perspectives on life, needs, and personal meanings. This structure allowed for the exploration of both individual and social/contextual factors associated with substance use and criminal behavior. Within the responsibility-attribution dimension, participants were invited to rate, on a scale from 1 to 10, the degree of responsibility attributed to various possible sources (family, school, peer group, community, society, self); however, this item was used solely as a qualitative elicitation tool—a starting point to prompt participants to explain and justify, in their own words, their attribution of responsibility—and not as a validated psychometric measure. Accordingly, the numerical scores were not analyzed or reported quantitatively; only the narrative content generated by the follow-up question (“Can you explain why you assigned these levels of responsibility?”) was integrated into the thematic analysis presented in Section 3.4. Prior to its use in the main study, the interview guide was pilot tested with approximately five incarcerated men meeting the same eligibility criteria; their responses were not included in the final analytic sample of 50 participants. The interview guide is presented in Appendix A.

Due to restrictions imposed by the prison environment, audio recording was not permitted, in accordance with the security rules applicable within the detention facility and with the regulations of the National Administration of Penitentiaries governing the conduct of research activities. Data were therefore recorded in full written form by the researcher and subsequently organized for analysis. To increase data accuracy, the researcher reread and completed the notes immediately after each interview. Following completion of the interview, the recorded content was discussed with the participant to confirm the accuracy of the information (member checking [32,33]), thereby reducing the risk of loss or distortion of relevant information. The limitations of this procedure, specific to the detention context, are discussed in Section Limitations.

In the absence of audio recording, the excerpts presented in Section 3 represent written reconstructions as close as possible to the participant’s own expression, recorded during or immediately after the interview and subsequently confirmed with the participant through member checking, rather than verbatim, word-for-word transcriptions of an audio recording. This distinction is relevant for interpreting the precision of the quoted statements and is discussed further in Section Limitations.

To visually distinguish these reconstructions from conventional verbatim quotation throughout this manuscript, all participant excerpts in Section 3 are set in italics, marking them explicitly as reconstructed statements rather than audio transcriptions; this formatting convention applies uniformly and is not repeated at each individual quotation.

### 2.4. Data Analysis

Data were analyzed using thematic analysis, following the steps described by Braun and Clarke [34]: familiarization with the data, generation of initial codes, identification of themes, review and refinement of themes, and final definition and naming of themes. To enhance the rigor of the analytic process, data coding was initially performed by the first researcher, who also conducted the interviews; a second researcher subsequently reviewed and coded the organized dataset, and discrepancies were resolved through discussion until consensus was reached. Because the second coder worked from data already structured by the first coder rather than from unsorted raw material, this process is characterized as consensus-based double coding rather than fully independent coding; this distinction is discussed as a limitation affecting the confirmability of the analysis in Section Limitations. Coding was performed manually, without dedicated qualitative data analysis software, consistent with the handwritten nature of the underlying interview notes (Section 2.3). Thematic saturation was actively monitored throughout the analytic process and was considered to have been reached when subsequent interviews no longer generated new codes or themes relevant to the research questions [35]. Some accounts simultaneously contained elements of the perceived effects of use and of justification for addictive behavior; in such cases, classification under one theme or another was based on the dominant emphasis of the narrative, established by consensus between the two researchers.

To ensure an explicit link between the identified themes and the study’s theoretical framework, themes were mapped a posteriori onto the recovery capital domains described by Cloud and Granfield [20]—physical, human, social, and cultural—without this mapping constraining the inductive coding process a priori (Table 1). It should be noted that Cloud and Granfield [20] conceptualize recovery capital as an interval-level variable situated on a continuum with positive and negative polarity, where zero is not the point of origin but a point along this continuum. Accordingly, the themes describing the effects of active use, withdrawal, and the mechanisms sustaining addictive behavior (3.1–3.4) were mapped onto the corresponding domains as manifestations of depleted recovery capital (the negative pole of the continuum), rather than as resources per se. Only themes 3.5 and 3.6 capture recovery capital with a predominantly positive or ambivalent polarity, representing resources that can be effectively mobilized in the recovery process.

**Figure 1:**
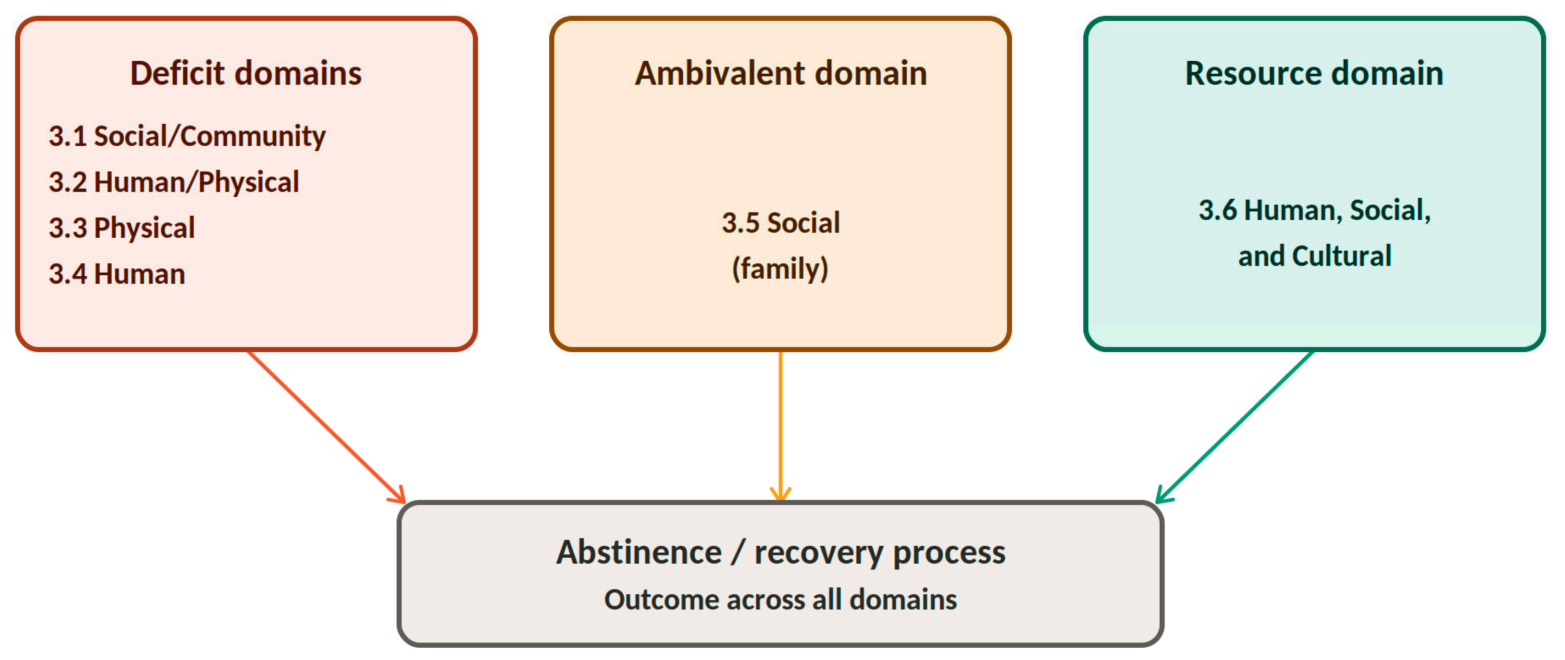
Conceptual model linking the six identified themes, grouped by recovery capital domain and polarity, to the abstinence and recovery process (see Table 1 for full theme–domain correspondence).

The polarity labels used in Table 1 are defined as follows, consistent with the continuum described by Cloud and Granfield [20]: “deficit (negative capital)” denotes a depletion or absence of resources within a given domain, situated on the negative pole of the continuum; “ambivalent” denotes a domain that functions simultaneously as a resource and as a source of depletion, as observed for family support in this sample; “resource (positive, emergent capital)” denotes assets actively mobilized, or beginning to be mobilized, in support of recovery (Figure 1).

### 2.5. Trustworthiness and Rigor

To ensure the quality of the research, the criteria of credibility, transferability, confirmability, and dependability were applied, as introduced by Lincoln and Guba [32] as an alternative to rigor criteria specific to quantitative research, and operationalized within the specific context of thematic analysis by Nowell et al. [36]. Data analysis was conducted iteratively, through successive revisions of codes and themes [37].

Interviews were conducted by one of the researchers (female), a specialist psychologist within the Directorate of Social Reintegration of the National Administration of Penitentiaries, with direct experience in the prison environment. This institutional affiliation facilitated access to participants, understanding of the carceral context, and the establishment of a trust-based working relationship. This same researcher performed the initial coding of the written notes. A second researcher, who had not participated in the interviews and had no prior knowledge of the first researcher’s interpretations, subsequently reviewed and coded the organized dataset. Because the second coder worked from data already structured by the first coder rather than from unsorted raw material, this constituted consensus-based double coding rather than fully independent coding, a distinction discussed as a limitation in Section Limitations. Comparing and discussing the codes until consensus was reached (Section 2.4) constituted the main mechanism for counterbalancing the partial overlap between the interviewer role and the coder role. The potential influences of this dual affiliation—institutional and role-based within the analytic process—were further managed through the systematic documentation of reflexive observations after each interview, intended to distinguish participants’ accounts from the researcher’s interpretations. Although the stages of the analytic process followed the classic structure described by Braun and Clarke [34], researcher reflexivity was treated as an active and continuous component of the analysis, in line with the emphasis placed by Braun and Clarke [38] on the interpretive role of the researcher in qualitative research.

### 2.6. Ethical Considerations

The study was approved by the Ethics Committee of the “Constantin Rădulescu-Motru” Institute of Philosophy and Psychology, Romanian Academy, Bucharest (no. 79/19 March 2021), and by the National Administration of Penitentiaries (no. 253715/23 September 2021).

Participants signed informed consent forms after being informed about the purpose of the research, the voluntary nature of participation, and the right to withdraw at any time. All data were anonymized to protect participants’ identity and ensure the confidentiality of the information provided.

As part of this consent procedure, participants were explicitly informed that no information they provided would be used for legal or institutional decision-making concerning their case (see Appendix A.2). This assurance was communicated verbally at the outset of each interview, alongside the written consent process; no additional, formalized protocol for reinforcing this message during the interview itself was documented, and the extent to which this fully mitigated the institutional authority differential inherent in the interviewer’s role could not be independently verified. This is addressed further as a limitation below.

## 3. Results

The studied sample consisted exclusively of men with a history of illicit drug use. Prior to incarceration, 74% of participants came from urban areas and 26% from rural areas. The age-group distribution showed that 32% of respondents were between 20 and 30 years old, 52% between 31 and 40, 14% between 41 and 50, no participant fell within the 51–60 range, and 2% were between 61 and 70 years old.

Regarding civil status, most participants were in a cohabiting relationship (52%), followed by unmarried individuals (32%), married individuals (10%), and divorced individuals (6%). Regarding the main type of illicit drug used, 40% of respondents reported marijuana use, 30% heroin use, 16% cocaine use, and 14% use of new psychoactive substances (“legal highs”).

In addition, more than half of participants reported having, over their lifetime, used at least two of the substance categories mentioned, suggesting a history of polydrug use.

For clarity, the sociodemographic and substance-use characteristics reported above are summarized in Table 2.

The thematic analysis identified six overarching themes, each comprising several related subthemes. These themes are presented below and illustrated with representative quotations from the participants.

### 3.1. Initiation Factors and Vulnerabilities Associated with the Onset of Illicit Drug Use

This theme describes the social, economic, and relational circumstances that favored first contact with illicit drugs.

The data indicate that the onset of psychoactive substance use predominantly occurs during adolescence and young adulthood.

A proportion of 44% of participants reported initiating use of illicit substances between the ages of 16 and 20, 28% between 21 and 25, 20% between 10 and 15, and 8% between 30 and 35.

Thematic analysis of the interviews shows that the initiation of use is strongly socially contextualized, being influenced by the peer group, the residential environment, and the accessibility of substances. Most commonly, onset occurred in informal contexts within the peer group, particularly in residential neighborhoods, where use was normalized and associated with social belonging.

(a) Peer group influence and the need for belonging

*“I only used one drug, that being weed. I was with a group of friends, at my place. We were playing a shoot-‘em-up game on the computer. Being high, it was like I had more dexterity and I aimed a lot better”.*

(marijuana user, 26 years old)

*“In 2020, at a party, I found out my partner was using cocaine, and she pulled me into a group of friends who used, which got me using too, so I’d be accepted into that circle of friends”.*

(heroin user, 30 years old)

*“The first time I used legal highs was at school, with my classmates”.*

(new psychoactive substance user, 27 years old)

(b) Exposure to use in criminogenic environments

*“I was under arrest for theft, and I got high for the first time in prison, together with a cellmate who had some. He suggested we get high together, because he didn’t want to hit the stuff alone, telling me I’d feel really good and forget all my problems. That’s how it started”.*

(marijuana user, 20 years old)

(c) Economic vulnerabilities and drug accessibility

*“I started using because of the pretty bad financial situation I was in. I started selling heroin, and then, out of curiosity and not knowing much about using, I started using it myself too”.*

(heroin user, 20 years old)

### 3.2. Clinical and Psychological Manifestations of Drug Use

This theme reflects the perceived effects of use on emotional, cognitive, and behavioral functioning.

(a) Euphoria, disinhibition, and reduced psychological distress

*“The moment I got high, besides feeling good, I just stopped caring about anything. I wasn’t scared, and I felt like the whole world was mine. I had no inhibitions”.*

(cocaine user, 25 years old)

*“A state of detachment from everyday worries and problems, which caused me depression and insomnia during the hard times in life”.*

(new psychoactive substance user, 25 years old)

(b) Reinterpreting use as a positive experience

*“The experience is different from one state to another. I’m very different when I’m high—I take really good care of my kids, I play with my kids, I do everything I can to see them happy”.*

(heroin user, 47 years old)

(c) Deterioration in health and social functioning

*“After a long period of using, I just had no energy for anything anymore. I’d forget things, I had no appetite, I slept a lot, I was irritable, I had no interest in sex anymore, I had to drag myself to get to certain important places. My connection with my family left something to be desired, I started closing myself off and smoking a lot. Even as I watched myself sinking deeper into my thoughts, I didn’t have the strength to stop”.*

(marijuana user, 31 years old)

### 3.3. The Experience of Dependence and Withdrawal

Participants described withdrawal as a complex experience, with severe physical and psychological symptoms.

(a) Intense physical symptoms

*“I don’t even want to remember it. I hated the vomiting spells. I’d throw up some yellow, bitter stuff. That happened when I didn’t get my dose on time. I never want that again”.*

(new psychoactive substance user, 40 years old)

*“Very hard to put into words. Bone pain, vomiting, heavy sweating, insomnia. Your eyes water and your legs just give out”.*

(heroin user, 40 years old)

(b) Sensory and neuropsychological manifestations

*“It was like a high-voltage electric current started right in the middle of my brain and went down through the middle of my bones. It mostly happened to me while I was sleeping”.*

(cocaine user, 40 years old)

(c) Denial of dependence and minimization of effects

*“I never had withdrawal. Anyway, marijuana’s a soft drug, it doesn’t do you any harm. I just slept more and didn’t feel like doing anything”.*

(marijuana user, 28 years old)

### 3.4. Cognitive and Social Mechanisms Sustaining Use

This theme captures how participants explain, justify, or sustain addictive behavior.

(a) Assuming personal responsibility

*“It’s my fault. I was told that if I liked it I’d get hooked and keep using, but I took it as a joke. I’m the only one to blame, not my family or my friends… they told me what could happen… my family told me to quit, but I kept lying to them, because by then I was already hooked”.*

(new psychoactive substance user, 31 years old)

(b) Attributing responsibility to external factors

*“My friends’ fault is that they influenced me. They told me you feel good and nothing bad happens to you”.*

(cocaine user, 29 years old)

*“I ended up using drugs because of society as a whole… you can find them really easily and fast, and they get sold to anybody”.*

(marijuana user, 23 years old)

*“I don’t feel guilty about it, because I’m not the one to blame. It’s their fault for corrupting me”.*

(new psychoactive substance user, 42 years old)

(c) The dependence–criminality cycle

*“I’d commit crimes to buy more, so I could get high and make some money too”.*

(heroin user, 34 years old)

*“At first I had money from my job and from my family, then I started borrowing. As a last resort, when I had no way left to get money, I turned to stealing”.*

(marijuana user, 29 years old)

*“My parents were always really rich. I never lacked for anything materially. They didn’t even know what I was doing with the money”.*

(marijuana user, 23 years old)

### 3.5. Family: Risk Factor, Protective Factor, and Source of Suffering

The family appears across all stages of use, from onset to recovery.

(a) Family models and upbringing experiences

*“I come from what I’d call a normal family. I got a good upbringing. They taught me right from wrong. Unfortunately, coming from a modest family, my folks were focused on work, and I ran wild until there was nothing more they could do about it”.*

(marijuana user, 34 years old)

*“They were too soft raising me. They never scolded me, no matter what stupid thing I did”.*

(marijuana user, 37 years old)

*“My parents were always stricter, but that wasn’t enough to stop me from ending up using drugs”.*

(marijuana user, 42 years old)

(b) Family suffering caused by addiction

*“I feel guilty that I didn’t spend more time with my kids and that lately I’ve only been in and out of prison… that’s brought me a lot of suffering… I lost my family, I lost my partner, the mother of my two kids. I lost some really important years of my life”.*

(new psychoactive substance user, 50 years old)

*“The hardest thing to bear was seeing the suffering in my parents’ eyes. My mother’s heart will never be the same again. As for my father, I think deep in his soul he’ll never forgive me… I made him a laughingstock in front of everyone”.*

(cocaine user, 29 years old)

*“I deeply, deeply regret how badly I treated my kids and my wife. I took the last money from the house to buy drugs. I never thought I was leaving them without food. I was never with them at kindergarten, I never went to their school shows. I wasn’t a good father… or a good husband either”.*

(new psychoactive substance user, 31 years old)

### 3.6. Recovery, Abstinence, and Identity Reconstruction

This theme describes the factors that favor or hinder recovery.

(a) Ambivalence toward quitting

*“I never wanted to quit, up until now, but now I’d like to quit for good. I’m getting on in years, I’m sick of this world. It’s been seven months since I’ve been locked up and I’m clean. My mind drifts to drugs every day, especially when someone’s talking about them”.*

(heroin user, 37 years old)

*“I’ve never felt guilty about it. I don’t see why I’d quit drugs, because either way, I don’t act ugly when I’m high”.*

(heroin user, 35 years old)

(b) Persistent positive beliefs about drugs

*“Drugs help me see the world differently… to be more sensitive and understand what love really means. I understood the true meaning of literature and romanticism. Drugs help you see the world from above”.*

(marijuana user, 23 years old)

*“My advice would be to only use cocaine, because the rest of the drugs are really dangerous and put your life at risk. That is, if they had no other choice… cocaine isn’t addictive… personally, I wouldn’t quit”.*

(cocaine user, 42 years old)

*“If you’re an artist and you really want to put on a show, it’s worth using. That’s the only way I managed to have my best performances. You’ve got energy and you’re creative… the whole world is yours and you put that across. You’re free to express whatever you want”.*

(cocaine user, 37 years old)

(c) Recovery and protective resources

*“The solution for getting away from drugs is having the ambition to leave behind the crowd you used with, to start a family and go to work, to mind your own business”.*

(cocaine user, 47 years old)

*“Educating young people. When I got into drugs, I didn’t know anything about them”.*

(heroin user, 50 years old)

*“Family is the only one who truly wants what’s good for you and can really help you. My family never left me on my own. My parents took me to a center, to get away from drugs”.*

(cocaine user, 29 years old)

*“Not to listen to what others tell them, to listen to their family, because they’re the only ones who truly want what’s good for them, and to never touch drugs”.*

(marijuana user, 31 years old)

*“To cut off all contact with the crowd that uses, or if they don’t have a crowd and use alone, to think about what their greatest wish on earth is. To realize they’re on a bad path and that everything depends only on them”.*

(marijuana user, 27 years old)

## 4. Discussion

The present study makes an original contribution to the literature on substance use disorders in vulnerable populations from three complementary perspectives. First, it provides qualitative data from a national context—Romania and Eastern Europe—that is underrepresented in the international literature on recovery capital and post-incarceration reintegration, where research is dominated by studies from Western Europe and North America. Second, it integrates within a single analytic framework dimensions that are typically studied separately: initiation of use, mechanisms of maintenance, the experience of withdrawal, the role of the family, and perspectives on social reintegration, offering a comprehensive picture of the addictive trajectory within the prison context. Third, the findings highlight the specific nature of institutionally constrained abstinence within the prison environment as distinct from internally motivated recovery—without implying that constrained abstinence is inherently devoid of therapeutic value—with direct implications for the design of therapeutic interventions and health policies addressing this population.

The remainder of this section is organized around four central contributions: (1) initiation and the environmental/social determinants of use; (2) the psychobehavioral trajectory of use and participants’ attribution of responsibility; (3) the family as an ambivalent recovery-capital resource and the distinction between institutionally constrained abstinence and clinical recovery; and (4) implications for social reintegration and continuity of care.

### 4.1. The Experience of Use and Early Onset

The study findings indicate that the onset of psychoactive substance use is predominantly, though not exclusively, concentrated in adolescence and early young adulthood: 20% of participants report onset between 10–15 years and 44% between 16–20 years—the category representing the most frequent initiation window in this sample—consistent with, though not itself proof of, the heightened vulnerability documented for this developmental stage in the wider literature. However, a significant proportion of the sample (28%) reports initiation between 21–25 years, and a minor subgroup (8%) between 30–35 years, suggesting that the onset of use in this sample is not a phenomenon confined to adolescence but extends, for a substantial part of participants, into early adulthood.

The vulnerability of adolescence to substance use initiation is well documented in the international literature, explained through the combination of incomplete prefrontal cortex neurodevelopment and the heightened social pressure specific to this stage [2,39,40,41]. The present study’s data are consistent with, and extend, this perspective by showing that in the Romanian context early onset is closely linked to the normalization of use within the peer group and the accessibility of substances in urban residential environments.

Trends toward earlier onset of substance use are reported globally, with recent studies highlighting increasingly earlier involvement of adolescents in opioid-related behaviors and an elevated risk of developing opioid use disorders when initiation occurs at a young age [42].

An important finding of the study is the predominantly social character of initiation. Participants describe onset occurring in informal contexts, within the peer group, in residential settings, or in recreational environments, where use is normalized. These findings are consistent with the literature emphasizing the role of social networks in the initiation of use behaviors [39,43,44].

Illicit drug use within the prison setting is another important aspect. According to available studies, it is not an isolated behavior; it has been documented in numerous European and non-European countries. Montanari and colleagues [18], in a study of 12,918 incarcerated individuals across seven European countries, found that reported illicit drug use levels were higher than in the general population but lower than those recorded prior to incarceration. Austin, Favril, Craft, Thliveri, and Freeman [26] show in their systematic review that incarcerated individuals use drugs during imprisonment, and that use within the prison environment is associated with multiple negative health consequences and with criminal recidivism. At the same time, some individuals initiate use while incarcerated.

The presence of onset within institutional settings (school and prison) extends the classic understanding of risk contexts and is consistent with the concept of the “risk environment,” which explains how structural and contextual factors influence use even within restrictive settings [45].

### 4.2. Psychobehavioral Trajectory of Use and Attribution of Responsibility

In this sample, participants’ accounts indicate a dual dynamic of use: initially positively perceived effects and progressive negative consequences. Immediate effects include euphoria, reduced anxiety, and increased confidence, suggesting a dysfunctional emotional regulation mechanism.

It should be clarified that participants’ accounts describe subjective experience, not neurobiological process; the dopaminergic mechanism proposed below is drawn from the external neuroscience literature as a candidate explanatory framework for these accounts, not a finding directly measured or observed in the present qualitative dataset. Neurobiological models of addiction offer a coherent explanatory framework for these findings: activation of the dopaminergic reward system and the reduction in emotional discomfort through use create a negative reinforcement pattern that sustains addictive behavior beyond voluntary intent [7,46]. The emotional regulation difficulties identified in participants’ narratives are consistent with recent clinical literature, which associates them with maladaptive coping strategies, including antisocial or impulsive behaviors, as well as with greater severity of substance use disorder [47]. In the specialized literature, individuals with substance use disorders are described as presenting persistent emotional regulation difficulties beyond cognitive awareness of negative consequences, an aspect that contributes to the maintenance of addictive behavior and to increased vulnerability to relapse [48]; the pattern reported by participants in the present study is consistent with this description.

Over the long term, participants describe cognitive deterioration, social impairment, and behavioral withdrawal, accounts compatible with the theoretical conceptualization of the transition from recreational use to a pattern of use marked by progressive loss of control. These findings are compatible with the literature showing that chronic substance use is associated with progressive deterioration in psychosocial functioning [49].

The variability in emotional and behavioral responses, including impulsivity and changes in empathy, supports the idea that the effects of use are substance- and context-dependent, an aspect also highlighted in the neuropsychiatric literature [7].

The data indicate that withdrawal is a severe, multidimensional experience, including intense physical symptoms (pain, vomiting, insomnia), neurological manifestations, and psychological instability.

The severity of withdrawal described by participants reflects the neuroadaptive nature of addiction as a chronic disorder of the reward and inhibitory control systems [7,46]. From a clinical perspective, this experience has direct implications for therapeutic practice within the prison setting. Although Romania has infrastructure dedicated to detoxification within the prison system, including hospital units with specialized detoxification wards, participants’ reported experiences indicate that untreated or insufficiently medically managed withdrawal amplifies craving and significantly reduces the capacity for voluntary abstinence. This discrepancy between the formal availability of services and the subjective experience of incarcerated individuals is consistent with recent specialized literature, which highlights that the effective use of medical services within the prison environment is a more reliable indicator of real accessibility than their mere formal existence [50]. These findings underscore the need to assess effective access, the timeliness of intervention, and the perceived quality of supervised detoxification protocols, rather than their formal existence alone.

A notable pattern was the normalization of absent withdrawal and the denial of dependence, particularly regarding cannabis. This type of cognitive reinterpretation reduces risk perception and may constitute a barrier to accessing treatment, an aspect frequently reported also in the literature on cognitive distortions in addiction.

Closely related to this psychobehavioral trajectory is how participants made sense of it retrospectively—in particular, how they attributed responsibility for the onset and maintenance of use.

The interview findings reveal a polarization in discourses concerning responsibility for illicit substance use, with participants oscillating between assuming personal responsibility and attributing use to external factors such as the peer group or society. These findings reflect the existing debate in the specialized literature regarding the status of addiction as a medical disorder versus choice-influenced behavior—a polarization also confirmed empirically at the level of public perception: a recent experimental study showed that most respondents attribute addiction to both a disease component and a personal-choice component, and that presenting official medical positions supporting the disease model does not significantly change perceptions regarding the criminal responsibility of individuals with substance use disorders [51].

By contrast, Heyman [52] argues that the maintenance of use is also influenced by voluntary decision-making processes, in which immediate rewards are preferred over long-term benefits. The coexistence of these perspectives within participants’ discourses suggests that attribution of responsibility is a complex process, situated at the intersection of neurobiological vulnerability, psychosocial factors, and individual self-regulatory capacity—an aspect with important implications for therapeutic interventions and recovery strategies.

### 4.3. Family as Ambivalent Recovery Capital and the Boundary Between Institutional Abstinence and Recovery

In this sample, the family appears to function simultaneously as a risk factor and a protective factor in the development of use behaviors. Family support is largely described by participants as the main protective factor, which is consistent with the recovery capital literature, which highlights the role of social capital in maintaining abstinence [19,20,21,53]. However, the data show important variability in the quality of family support (support, permissiveness, lack of involvement), suggesting that the family does not function uniformly as a protective factor.

Recent literature identifies low parental monitoring, poor communication, and limited family involvement as consistent predictors of the initiation and maintenance of substance use among adolescents and young adults [54,55,56]. The present study’s data nuance this relationship, showing that the protective effect of the family is neither automatic nor uniform—the quality of support, not merely its presence, appears to determine the difference between a family environment that is protective and one that indirectly facilitates use through permissiveness or inadequate supervision.

The present study’s data also suggest that economic vulnerability can amplify the risk of use and of involvement in criminal activities to obtain substances. These findings support Goldstein’s [57] economic-compulsive model, according to which addiction and criminality can become interdependent in the context of limited financial resources and social marginalization. These processes are also supported by recent reports highlighting the persistence of socioeconomic inequalities and social exclusion as structural factors associated with problematic substance use and related criminality [25]. For incarcerated individuals, the dynamics of family support take on a particular form, being influenced by the frequency of visits, the quality of relationships maintained during incarceration, and the continuity of emotional bonds. While some families continue to provide emotional and material support throughout the sentence, participants associated this continuity with sustained motivation for change, though the cross-sectional qualitative design does not permit inferring an effect on recidivism; other situations are marked by progressive abandonment, which participants linked to heightened psychological vulnerability, feelings of social exclusion, and deterioration in mental health.

From a medical and psychosocial perspective, the lack of consistent family support during the post-incarceration period is frequently associated with reintegration difficulties, lower treatment adherence, and increased risk of relapse into substance use or worsening of pre-existing psychiatric disorders. The transition from the prison environment to the community thus represents a critical stage, in which continuity of medical care, access to mental health services, and the existence of a social support network can significantly influence long-term outcomes. In the absence of these resources, released individuals may experience heightened anxiety–depressive symptomatology, adjustment difficulties, and an increased risk of social marginalization, underscoring the need for integrated interventions centered on both the family component and the medico-social component.

Family support was a resource participants repeatedly associated with periods of abstinence, which raises the question of what these episodes of abstinence actually represented—a question addressed directly below.

In this sample, abstinence appears to be an unstable process, shaped by the interaction of individual, social, and institutional factors. Two main factors are identified: family dynamics—which function both as a source of support and, through the ruptures and suffering associated with use, as a motivational turning point for change—and incarceration as a factor of institutional constraint.

A recent study conducted on an adolescent population confirms the co-occurrence of substance use and violent behaviors in contexts of social and family vulnerability, underscoring the cumulative role of individual and environmental factors in the initiation of risk behaviors [58].

Abstinence within the prison environment is frequently described by participants as forced rather than voluntary, accompanied by persistent craving. Procedurally, incarcerated individuals experiencing a withdrawal crisis are transferred to the prison hospital or infirmary, where their condition is medically monitored. They may also receive specific psychological assistance, depending on identified needs and available interventions, so that they are not left without supervision and support during this period. Nonetheless, the subjective experience reported by participants indicates that institutionally imposed abstinence does not equate to psychological recovery. Although both medical support and psychological interventions exist, participants’ accounts suggest that the process of withdrawal management and recovery support requires improvement to more adequately meet their needs. Individuals in periods of involuntary abstinence continue to face significant challenges, such as persistent craving, disadvantaged living conditions, and lack of social support, which make maintaining long-term abstinence difficult [59].

The findings also support the idea that recovery is a complex process, involving not only abstinence but also the restructuring of psychological and social capital [60,61].

The present qualitative data support the relevance of cognitive–behavioral and motivational approaches to the difficulties participants described (craving, cognitive distortions, ambivalence toward change), but do not themselves demonstrate the effectiveness of such interventions in this population; the recommendation below is grounded in the broader intervention literature rather than in outcomes observed in this study, and its implementation would benefit from dedicated intervention research within Romanian penitentiary settings. From a clinical perspective, therapeutic interventions based on cognitive–behavioral and motivational principles should be systematically integrated into assistance programs for incarcerated individuals with a history of substance use, aiming to reduce relapse vulnerability and facilitate the transition to sustained post-release abstinence. These interventions have proven effective by acting on the core mechanisms involved in substance use disorders, including cognitive distortions, inhibitory control deficits, and conditioned responses to use-associated stimuli [62].

From a neurobehavioral perspective, they contribute to modulating the reactivity of the dopaminergic reward system and to strengthening the executive functions involved in self-regulation, thereby reducing susceptibility to craving and compulsive use behaviors [63,64].

Within the prison population, frequently characterized by psychiatric comorbidities, socioeconomic vulnerability, and elevated risk of recidivism, such interventions should be complemented by motivational and psychoeducational components, adapted to the level of cognitive functioning and to the institutional context.

In addition, the spiritual dimension and the reconstruction of personal meaning around the illness experience can be integrated as adjuvant factors in the recovery process, being associated with better emotional regulation, increased resilience, and reduced risk of relapse, by facilitating post-addictive identity reconfiguration and narrative coherence [65].

### 4.4. Social Reintegration, Recidivism Risk, and Implications for Continuity of Care

According to the accounts of incarcerated individuals, attitude toward life is correlated with the attitude toward the addictive behavior that existed prior to incarceration. Thus, incarcerated individuals who did not report negative aspects related to illicit drug use also did not mention any intention to quit using.

The impact of substance use on the social reintegration process is significant, particularly for individuals with a history within the criminal justice system. A history of use affects reintegration capacity by interfering with employment, housing stability, interpersonal relationships, and compliance with social norms.

Continuity of care after release from detention is necessary, by connecting participants to medical, psychiatric, and social services. Strauss, Swerin, and Rodgers [66] showed that coordinated access to services reduces the risk of recidivism and improves housing stability and mental health. Accordingly, collaboration between the justice system and health service providers to support the effective community reintegration of vulnerable individuals is essential.

The findings highlight that the meaning of life is strongly restructured around the family, which becomes the primary moral and identity anchor. The loss of family relationships is perceived as one of the most significant consequences of use.

These findings are consistent with the literature showing that substance use profoundly affects social reintegration through its impact on interpersonal relationships, employability, and social stability [49,67].

Furthermore, the risk of recidivism is increased in the absence of post-release support, particularly among individuals with a history of use [10,17].

The predictive validity of the risk–needs–responsivity model in assessing recidivism risk is supported meta-analytically, with studies confirming that interventions targeting dynamic criminogenic factors—including substance use—significantly reduce the likelihood of reoffending when adapted to the individual’s risk level and needs [68].

A relevant aspect is the ambivalence of discourses: narratives of reconstruction (family, work, education) coexist with narratives normalizing use, reflecting the internal conflict between change and the maintenance of addictive behavior.

Double stigmatization (former inmate + substance user) represents an additional barrier to reintegration, limiting access to social and professional resources [22,69].

From a medical and public health perspective, “attitude toward life” among individuals with substance use who are incarcerated or post-release is not treated as a moral trait but as a modifiable clinical construct, influenced by addiction, psychiatric comorbidities, chronic stress, and social exclusion. Reducing depressive symptomatology and demoralization, increasing self-efficacy, and reconstructing a functional sense of meaning in life are necessary, as these factors can improve adherence to abstinence and reduce the risk of relapse.

From a public health and addiction medicine perspective, the findings support the need to implement integrated continuum-of-care programs for individuals with a history of substance use undergoing post-release social reintegration, including clinical management of the substance use disorder, psychiatric monitoring, structured psychosocial interventions, and occupational support. These interventions should be oriented toward reducing relapse risk by strengthening treatment adherence, improving psychosocial functioning, and restoring social support networks, particularly family networks, as well as by addressing the structural factors associated with social vulnerability and stigmatization.

## 5. Conclusions

This study, conducted on a sample of 50 men from a single penitentiary in Romania, shows that the use profile reported by participants is consistent with the conceptualization of psychoactive substance use as a phenomenon with a chronic, multifactorial course and biopsychosocial determination, with early onset reflecting heightened neurodevelopmental vulnerability and early exposure to high-risk social environments. The presence of initiation within institutional contexts, including school and prison, extends the classic understanding of risk factors and is consistent with the “risk environment” model, in which structural determinants influence addictive behavior even within restrictive settings; further data, from a broader range of prison contexts and including incarcerated women, are needed to assess the extent to which these findings extend beyond the studied sample.

The data suggest that participants perceived addiction ambivalently, attributing it to both individual and external factors; this ambivalence is consistent with—though it does not directly test—the literature describing addiction as involving neurobiological alterations in reward and executive-control circuits alongside psychosocial determinants. From a clinical perspective, this profile supports the need for integrated interventions that move beyond the choice–disease dichotomy and include medical management, psychological interventions, and structured social support.

The evolution of use follows a progressive pattern, from acute reinforcing effects (euphoria, anxiolysis, increased perceived self-efficacy) toward cognitive, affective, and social deterioration. Withdrawal emerges as a central element in sustaining the cycle of dependence and relapse, being associated with severe symptomatology and persistent craving, an aspect consistent with the neuroadaptive model of addiction described in the literature [70].

The normalization of use and the denial of dependence constitute significant barriers to accessing treatment services.

In this sample, the family represented an important determinant with a bidirectional role, functioning both as a protective factor through social capital and emotional support, and as a risk factor in the context of relational dysfunction or inadequate supervision. In parallel, socioeconomic vulnerability contributed to the maintenance of problematic use and its association with criminal behaviors, consistent with economic-compulsive models of addiction.

In this sample—selected precisely for the capacity to sustain short-term abstinence (Section 2.2)—abstinence within the prison environment was frequently perceived by participants as constrained rather than as equivalent to clinical recovery, which involves psychological, behavioral, and social restructuring. The recovery process is unstable and dependent on the interaction of individual, relational, and institutional factors, being decisively influenced by the continuity of post-release support.

Because participants were interviewed while still incarcerated, post-release outcomes were not directly observed in this study; based on the broader literature and on participants’ own anticipations regarding release, the literature on post-release reintegration suggests that the lack of continuity of care, combined with stigmatization, psychiatric comorbidities, and the fragility of social networks, may increase the risk of relapse and recidivism [49,67]—an association this study’s cross-sectional, pre-release data cannot itself establish. In this context, social reintegration must be understood as an integrated clinical and social process, not merely as legal reinsertion.

From a public health and addiction medicine perspective, these findings point toward continuum-of-care models adapted to this vulnerable population, detailed in the three recommendations below.

The three recommendations below are informed by the present findings but extend beyond what a single qualitative study of 50 men in one penitentiary can establish; they should be read as directions warranting policy consideration and dedicated evaluation research, not as effects demonstrated by this study. First, the findings of the present study have direct practical implications for the healthcare system and public policy in Romania. Official EUDA data confirm the existence of relevant institutional measures within the Romanian prison system, including methadone opioid agonist treatment (OAT) and psychological programs for individuals with substance use disorders. Nonetheless, the findings of the present study support the need to strengthen and standardize these measures within a coherent national framework of therapeutic intervention, similar to the recommendations formulated by EUDA for European Union member states [25], given that existing services remain, according to participants’ reported experiences, fragmented and unevenly distributed across prison units.

Second, the findings underscore the need to develop continuum-of-care programs that ensure continuity of care from the moment of incarceration through post-release community reintegration. These should include clinical management of the substance use disorder, evidence-based psychotherapeutic interventions—particularly cognitive–behavioral and motivational approaches—psychiatric monitoring of comorbidities, and structured occupational support. The recent reorganization of the national anti-drug system through the dissolution of the National Anti-Drug Agency and the creation of a new structure directly subordinate to the Prime Minister [71] represents an opportunity to reconfigure these services with a clearer orientation toward public health rather than internal order.

Third, given the central role of the family identified in the present study, prison-based interventions should systematically include family involvement components—family counseling, facilitation of visits, and the maintenance of emotional bonds during incarceration—recognizing the family not merely as an informal resource but as an active therapeutic element in the recovery process. Structured collaboration among the prison system, mental health service providers, and community organizations may represent an important lever for reducing the risk of relapse and recidivism in the post-release stage, though this study cannot itself demonstrate such an effect.

### Limitations

One limitation of the study relates to the institutional restrictions of the prison environment, which did not permit audio recording of the interviews. Manually recorded data carry an inherent risk of losing certain nuances of verbal communication, a risk mitigated through immediate note-verification procedures and confirmation of the accuracy of the information together with the participant. Consequently, the excerpts presented in Section 3, although rendered in quotation marks and validated through member checking, must be interpreted as written reconstructions of participants’ discourse, as faithful as possible, rather than as verbatim transcriptions from an audio recording; the exact wording, hesitations, or prosodic nuances of the original discourse may not have been captured with the same precision as in a classic verbatim transcription.

Beyond the loss of prosodic nuances, the absence of audio recording raises a more substantial limitation: the fidelity of the content of the accounts, not merely their form, depends on the accuracy of manual note-taking performed by a single researcher, in real time or immediately after the interview. Although the member-checking procedure was applied systematically, it took place orally, immediately after the interview, within the penitentiary premises, with the same researcher who had recorded the notes—conditions that differ from standard member-checking practice [32], which is typically conducted afterward, based on a complete transcript, and in a context that minimizes relational pressure between participant and researcher. Within the carceral environment, the power asymmetry inherent in the relationship between a participant and any person associated, even indirectly, with the detention institution may favor compliance or passive acceptance of the researcher’s proposed wording, rather than its contestation. This limitation must be taken into account when interpreting the accuracy of the narrative content presented in Section 3, not merely its linguistic form.

An important limitation of the study is the possible social desirability of responses, given that some participants showed a tendency to assume responsibility for illicit drug use or to construct their discourse in a morally acceptable manner, without entirely excluding the possibility of relapse or resumption of criminal behavior. This aspect may influence the authenticity of certain accounts, particularly regarding the real motivations underlying use and behavioral change.

Two further limitations concern the documentation of the recruitment and saturation procedures. First, the exact number of eligible individuals approached and the number who declined participation were not systematically recorded during the recruitment process; while the open-invitation, quota-based procedure described in Section 2.2 explains why a conventional recruitment flow was not tracked, the absence of this record limits the ability to characterize non-participation and should be acknowledged as a limitation in its own right. Second, thematic saturation was assessed through ongoing consensus between the two coders as coding progressed, rather than through a documented log tied to specific interview numbers; the absence of a more granular, interview-by-interview record of code emergence is acknowledged as a limitation of the saturation assessment procedure.

The retained coding notes do not permit a systematic quantification of how many participants contributed to each theme; consequently, qualitative frequency descriptors (e.g., “most,” “many,” “a minority of participants”) were not applied, and this is acknowledged as a limitation of the transparency of Section 3.

The family support theme is not analytically differentiated by sub-dimension (emotional versus instrumental support, partner versus parental versus children’s support, family involvement in prior treatment attempts, or family support before versus during incarceration); the retained coding notes do not support this level of granularity, and this coarser categorization is acknowledged as a limitation, particularly given the centrality of family support to the study’s objectives.

Although the sample’s distribution across substance categories and age groups is reported (Table 2), the coding notes do not support systematic disaggregation of themes by substance type or by age group; whether the trajectories, withdrawal experiences, or family dynamics described differ meaningfully across these subgroups could not be assessed in the present analysis and is acknowledged as a limitation. This heterogeneity, and its potential relevance to differentiated recovery pathways, is a priority for future research with a design supporting subgroup comparison.

Finally, because participation required a form of self-selection among eligible men (willingness to discuss substance use and incarceration with a researcher affiliated with the penitentiary system), the sample may under-represent individuals with particular motivations for declining or, conversely, for volunteering; the possibility that some participants’ accounts reflect impression management beyond the social-desirability concern noted above cannot be excluded and is acknowledged as an additional limitation of self-report data in this population.

Another limitation stems from the cross-sectional design of the research, which captures participants’ experiences at a single point in time, without allowing for tracking the evolution of abstinence, relapse, or social reintegration processes. For this reason, the temporal dynamics of change, as well as stable causal relationships among the variables explored, could not be captured.

A further methodological limitation stems from the inclusion criterion of self-reported abstinence of at least 30 days at the time of the interview. Although clinically justified by the need for reflective accounts unaffected by acute intoxication or active withdrawal, this condition introduced a systematic selection of the sample toward individuals capable of maintaining short-term abstinence within the prison environment, excluding individuals with active use or recent relapse during incarceration. Consequently, the central theme concerning the perception of abstinence as maintained under institutional constraint, rather than as internally motivated recovery, must be interpreted as specific to this selected subgroup, not as representative of the entire population of incarcerated individuals with a history of use in the studied penitentiary. Future research would benefit from including a broader sample, encompassing individuals with active use or recent relapse during incarceration, to assess whether the perception of abstinence as a constrained phenomenon persists in this subgroup as well, or reflects, at least in part, the specific characteristics of the sample selected for the present study.

Finally, the generalizability of the findings is limited by the sample characteristics: it consisted exclusively of men, selected through purposive (non-probability) sampling from a single penitentiary in Romania. The experiences of incarcerated women with a history of substance use, as well as any institutional particularities of other prison units within the country or region, are not captured by the present data. Consequently, the study’s findings must be interpreted as specific to this context and this sample, and their transferability to other populations or prison systems is to be assessed by the reader based on the similarity of contexts [32], rather than implicitly assumed.

In addition, the data are based exclusively on self-report, which carries the risk of distortions related to memory, retrospective reinterpretation of events, or selective omission of sensitive experiences. The absence of triangulation methods (e.g., observation or complementary clinical/prison records) limits the ability to verify the consistency of the information provided.

Furthermore, the responsibility self-rating item on a scale from 1 to 10 (Appendix A.3) was used exclusively as a qualitative elicitation tool and was not analyzed or reported quantitatively; the absence of psychometric validation for this item, together with the sample size, would not have allowed for a robust statistical interpretation of the scores, which is why the associated narrative content was integrated directly into the thematic analysis.

Future research would benefit from a longitudinal design that would allow the evolution of substance use, abstinence processes, and social reintegration trajectories after release to be tracked. Such an approach would facilitate the identification of critical moments of relapse risk, as well as the inflection points at which interventions have the greatest impact.

It would also be relevant to investigate in greater detail the mechanisms of resilience and recovery among individuals with a history of use and incarceration, with an emphasis on the factors that support the maintenance of abstinence under conditions of heightened social vulnerability. In particular, the role of the family, social networks, and institutional interventions could be analyzed comparatively, to better understand which types of support have a real effect on the reintegration process.

## Figures and Tables

**Table 1:** Correspondence between the themes identified through thematic analysis and the recovery capital domains [20], with indication of polarity.

Theme (Results)	Recovery Capital Domain [20]	Polarity
3.1. Initiation factors and vulnerabilities associated with onset	Social/Community	Deficit (negative capital)
3.2. Clinical and psychological manifestations of use	Human/Physical	Deficit (negative capital)
3.3. Experience of dependence and withdrawal	Physical	Deficit (negative capital)
3.4. Cognitive and social mechanisms sustaining use	Human	Deficit (negative capital)
3.5. Family: risk factor, protective factor, and source of suffering	Social	Ambivalent (resource and deficit)
3.6. Recovery, abstinence, and identity reconstruction	Human + Social + Cultural	Resource (positive, emergent capital)

**Table 2:** Sociodemographic and substance-use characteristics of participants (N = 50), summarizing the data reported in Section 3.

Characteristic	Value
Residence prior to incarceration	Urban: 74%; Rural: 26%
Age group	20–30 years: 32%; 31–40 years: 52%; 41–50 years: 14%; 51–60 years: 0%; 61–70 years: 2%
Marital status	Cohabiting relationship: 52%; Unmarried: 32%; Married: 10%; Divorced: 6%
Main substance used	Marijuana: 40%; Heroin: 30%; Cocaine: 16%; New psychoactive substances (“legal highs”): 14%
Age at initiation of use	10–15 years: 20%; 16–20 years: 44%; 21–25 years: 28%; 30–35 years: 8%
Polydrug use (lifetime, ≥2 substance categories)	More than half of participants

## Data Availability

The data generated and analyzed during this qualitative study are not publicly available because they contain sensitive information concerning incarcerated participants and are subject to ethical, confidentiality, and institutional requirements. Interviews were not audio-recorded due to institutional security regulations; instead, detailed written notes were produced and validated through member checking. Appropriately de-identified study materials may be considered for access upon reasonable request to the corresponding author, subject to case-by-case assessment and applicable ethical, confidentiality, and institutional requirements. Access is not guaranteed and materials will not be released where disclosure could compromise participant confidentiality or institutional security.

## Appendix A. Semi-Structured Interview Guide

### Appendix A.1. Study Context

This interview guide was developed for a qualitative descriptive study investigating experiences, perceptions, and recovery-related factors among incarcerated individuals with a history of illicit drug use prior to imprisonment.

The guide was designed to elicit rich, descriptive accounts of substance use trajectories, perceived determinants of drug initiation, abstinence processes, and subjective life experiences, consistent with a qualitative descriptive approach [28,29].

A. Socio-demographic and contextual information

- What is your age?
- What is your place of residence prior to incarceration (urban/rural)?
- What is your marital status?
- Can you briefly describe your educational and occupational background?

B. Substance use history and lived experience

- At what age did you first use drugs?
- What substances have you used?
- Can you describe the context in which you first used drugs (where were you, with whom, under what circumstances)?
- How long have you used drugs in total?
- How would you describe your experience while under the influence of drugs?
- How would you describe withdrawal symptoms and the experience of abstinence?

C. Attribution of responsibility and perceived determinants

- On a scale from 1 to 10, who do you consider responsible for your drug use initiation (family, school, peers, community, society, yourself)?
- Can you explain why you assigned these levels of responsibility?
- What role did your family play in your upbringing and life development?
- How did you obtain financial resources to sustain drug use?

D. Abstinence, recovery attempts, and change processes

- Have you ever tried to stop using drugs? If yes, what motivated you?
- What helped you remain abstinent during those periods?
- How many times have you attempted to quit drug use?
- What were the main losses or negative consequences of drug use in your life?
- What do you consider the most effective way to overcome drug dependence?
- What advice would you give to someone trying to stop using drugs?

E. Subjective life perspective and meaning-making

- What unmet needs have you experienced throughout your life?
- Do you experience feelings of guilt or remorse related to drug use? If yes, in what way?
- What is your biggest regret in life?
- What is your greatest source of joy or satisfaction?
- If you could change something in your life, what would it be?
- Please complete the sentence: “Life is worth living because…”

### Appendix A.2. Ethical Framing Within Interview Process

- Participants were informed that participation was voluntary.
- They were assured that they could refuse to answer any question.
- Confidentiality and anonymity were guaranteed.
- No information provided was used for legal or institutional decision-making.

### Appendix A.3. Notes on Use

This interview guide was administered in a flexible manner, allowing the interviewer to adapt the order and depth of questions according to participant responses, in line with qualitative research principles of openness and elicitation of rich, descriptive accounts.
